# How a community-led understanding of access and uptake barriers and enablers informs better vaccination programs

**DOI:** 10.3389/frhs.2023.1260400

**Published:** 2023-10-20

**Authors:** Perrykent Nkole, Francesca Alice, William Mutua, Lois Osei, Alexander Stoljar Gold, Lily Yang, Anna Wadzanayi Matendawafa, Tian Johnson

**Affiliations:** ^1^ The African Alliance, Johannesburg, South Africa; ^2^ Pandemic Emergency Readiness Lab, McGill University, Montreal, QC, Canada

**Keywords:** vaccines, immunization, inequality, Africa, COVID-19, human rights, community-led monitoring, accountability

## Introduction

Interventions to increase timely and equitable access to life-saving diagnostics and medicines for Africa must address the root causes of inequality and prioritize meeting communities at their point of need. Current donor funding for vaccination initiatives and voluntary licensing largely remains at the discretion of those in the global north, curtailing the agency of African countries, their pandemic preparedness, and the need for decolonization of research and development, i.e., free intellectual property provisions that benefit the pharmaceutical industry's profit over people's lives (1).

The most recent example of this schism is the COVID-19 pandemic. COVID-19 vaccine distribution was extremely inequitable from the outset, with low- and middle-income countries (LMICs) left waiting at the back of the global vaccine line. Despite the efforts of bodies like COVAX (2), who dedicated significant financial resources towards sending vaccines to LMICs, only 512 million or 4% of the 13.5 billion doses administered worldwide, were given to people in LMICs (most of which are in Africa) (3). Meanwhile, countries like Israel, the United States, Canada, the United Kingdom, and many others have administered multiple booster shots to their populations, making pharmaceutical companies billions (3). Additionally, the approval of second-generation COVID-19 vaccines that offer better protection against SARS-Cov-2 variants of concern such as Omicron and Delta, has added another layer of inequity, with countries in the global north procuring these vaccines. In contrast, less effective first-generation vaccines continue to be delivered to LMICs. This extreme inequality in vaccine access is due to the prioritization by vaccine manufacturers of bilateral deals with rich countries i.e., profit over the health needs of the global population—including African countries—and vaccine nationalism, where many global north countries stocked up on vaccine supplies even while others (mainly in the global south) had very limited supply.

While the supply of COVID-19 vaccines, tests and treatment on the continent may have increased since the initial global vaccination effort, challenges persist concerning the distribution and administration of these medical interventions in-country, i.e., getting them from the ports they arrive at, into communities to access in a timely and coordinated way. These range from the logistics of delivering diagnostics and medicines in rural areas (physical infrastructure and access) to inadequate cold chain equipment and protocols to mis/disinformation and hesitancy fueled by unverified information shared across social media and by prominent public figures (4).

Underpinning these specific challenges is the already overburdened, under-resourced healthcare systems across Africa, undermining the continent's ability to prevent, prepare for and respond to pandemics—those that came before COVID-19 and those that will come after.

A community-led approach that can identify the specific barriers to a proposed response, in this case, a successful vaccination program, identify and promote enablers to access, and pair this approach with local advocacy linked to civil society preparedness, has the potential to increase communities' sense of agency, build confidence and trust in state-designed health emergency response.

The current top-down approach, driven by international agendas, has undermined public trust in African governments' health responses and led to vaccine hesitancy, fueled by a lack of information and effective awareness interventions. This contributed to wasting essential medicines and further mistrust in public health responses and resourcing. More evidence is urgently needed from a community perspective, i.e., those who continue to be most affected, on how the COVID-19 pandemic impacted public health systems, and how governments prepared for and responded to COVID-19, including their vaccine rollout strategies.

## What could a community-led vaccination rollout look like?

In mid-2021, a concept for public health accountability, “Ports2Arms”, was developed. This project recognized that, even in the early days of the COVID-19 pandemic, global efforts to distribute vaccines to developing countries and monitor these through various public health tracking mechanisms had little oversight of the specific barriers and enablers on the ground to equitable vaccine distribution and uptake. Such tracking would ensure that vaccines reach ports and are distributed in a way that recognizes and can respond to the realities of already strained public health systems, significant disease burdens, geographically dispersed communities, and inequitable access to public goods and services (5). Ultimately, these warnings were borne out with gross vaccine Inequality still evident between developing countries in places like Africa, and the rest of the world (6).

Through a community-led monitoring (CLM) approach, Ports2Arms works with national partners using a combination of media monitoring (TV, radio, newspapers, websites and social media); COVID-19 information availability in communities; the extent to which civil society was given advanced notice of incoming vaccine shipments and could prepare communities for uptake; and the specific vulnerabilities of underserved and marginalized populations, including the LGBTIQ + community, sex workers, people who use drugs, people living with HIV, people in detention, adolescent girls and young women, older people, and those living with a disability, among others. A story-gathering process enabled qualitative data collection to support mapping COVID-19 cases and vaccine rates and bring a human face to the data and other narratives captured through media monitoring. A co-analysis process with affected communities who had participated in the data collection sought to inform evidence-based advocacy, which for the pilot, saw a series of radio talkback shows hosted in each community during the pilot. Through this kind of multi-layered community-led monitoring (CLM) process, Ports2Arms aimed to ensure that, by learning from the South African COVID-19 vaccination experience at the community level, African communities and governments can be better prepared for and respond to future pandemics, incorporating better implementation of associated vaccination programs.

## Monitoring barriers and enablers as potential sites for action

When the first COVID-19 vaccines became available, it brought hope for many devastated by the pandemic, its ongoing socio-economic effects, and its high death rate. The vaccines symbolized a solution to national and global struggles. However, over time, tensions arose regarding safety, accessibility, equitable distribution, and supply challenges. Specific barriers to vaccine distribution, as defined by the Ports2Arms project, include:

• *Supply chain bottlenecks:* any issue along the ports-to-arms pathway occurring on the supply side which prevents vaccine doses from becoming readily utilized for individuals willing to be vaccinated (e.g., cold chain issues, vaccines received close to expiry, lack of healthcare workers)
• *Health workforce:* In the context of the COVID-19 response, this refers to inadequate or non-existent training, discrimination of some population groups (people are turned away) or equipment shortages [e.g., syringes, personal protective equipment (PPE)].

Barriers to vaccine access and uptake include:

• *Vaccine hesitancy:* factors that prevent an individual from wanting to become vaccinated (e.g., lack of trust in the government, misinformation)
• *Vaccine access (poverty):* barriers that hinder an individual from getting vaccinated. This encompasses any structural or social inequities that exist that actively or passively exclude certain people or groups within a population (e.g., Transportation costs, technological barriers).

*Political instability, conflict, and unrest* due to the disrupting effects of the COVID-19 response and caused by events such as local/national elections; protests (service delivery or other) and civil unrest related to the impact of COVID-19 on livelihoods cuts across both distribution and access barrier definitions.

The specific enablers of vaccine distribution and access can be defined as:

• *Training and skills development:* any initiatives focused on developing vaccine competencies.
• *Community outreach:* efforts to take Vaccines to communities to minimize associated access costs and challenges.
• *Multisectoral collaboration:* coordinated efforts of various sectors/organizations of a community that combine their resources/knowledge to deliver and encourage vaccination (e.g., government working with businesses or communities to promote vaccination or create vaccination sites).
• *Community engagement:* initiatives that mobilize community members to encourage vaccine uptake within their communities (e.g., local leaders' engagement and community vaccination initiatives).
• *Accountable leadership:* competent, timely, and responsible leadership that creates effective structures/programs/plans that encourage vaccination of their constituencies (e.g., creating a national vaccine strategy, creating programs to improve vaccine access).
• *International collaborative efforts:* any assistance from international organizations/countries to improve a country's capacity for vaccine absorption (e.g., donation of ultra-cold freezers, healthcare worker training).
• *Use of technology:* examples of innovation in the digital and technology spaces to facilitate vaccine access.
• *Incentives:* examples of incentives to facilitate access to vaccines (e.g., cash to cover associated costs or food vouchers as an incentive).

This diverse set of barriers–– from vaccine hesitancy to lack of transportation––commonly hinders vaccination efforts, having the greatest ramifications in the countries suffering most from vaccine inequity. If these barriers are to be overcome, it is critical that they be well understood and that the approaches taken to mitigate them be clearly identified. Monitoring enablers or good practices also supports this by identifying local solutions that facilitate a more equitable response. Such community-led mapping of barriers and enablers allows countries and communities to use effective solutions and discard unhelpful approaches, based on the lived experiences of the communities they are meant to service—hence increasing the speed and number of vaccines that travel from ports to arms.

## Why community-led monitoring?

CLM approaches have been particularly critical in contexts with weak health systems, where communities, on the one hand, experience health system failings (lack of personal protective and other equipment or stockouts), stigma and discrimination, and inadequate infrastructure, and on the other hand, lack the structures and ability to raise grievances due to cultural norms, power imbalances, and fear of reprisal. Despite this, community actions have been critical in securing political will and funding for HIV research, prevention, care, and treatment (7) and the evidence of CLM having delivered benefits to communities through policy and practice change at the local and national levels is a key driver in the recent mainstream attention brought to the approach by global agencies such as PEPFAR, Global Fund and UNAIDS. This was perhaps triggered by the COVID-19 pandemic's highlighting the impact of high levels of treatment interruptions for people living with HIV, but also recognizes the way CLM can identify specific barriers to services (access and uptake) and facilitate evidence-based community engagement and risk communication strategies to build individual confidence and trust in government public health guidance. There is also recognition of its broader applicability, for example, in humanitarian situations—to improve preparedness and response (for future pandemics) (8)—and other challenging environments to monitor related societal and structural interventions, including improving the legal environment, Human Rights promotion and protection, or action against stigma and discrimination.

However, while communities have provided feedback on the quality of health service provision since the early days of the HIV epidemic, the gathering, collation, and use of such data has not always been systematic or widespread, resulting in an evidence gap in terms of its efficacy for other kinds of public health crises, such as pandemics. There is also a significant gap in understanding how underserved and vulnerable populations are particularly affected (9).

## Conclusion

In conclusion, in the context of the lessons learnt from COVID-19 pandemic, which continues to impact communities on the continent, there is an urgent need for future emergency vaccination programs to consider:

• how to ensure a more equitable emergency response that includes visible global manufacturing and distribution plans for vaccines, other medical interventions and technologies, and a regular public reporting mechanism with meaningful civil society oversight.
• improved accountability, including a commitment from global leaders and bodies, including Gavi, the African Union, Africa Centre for Disease Control and the World Health Organization, to ensure consistent, meaningful and independent access for civil society and community observers from the onset of widespread vaccination initiatives.
• addressing roots of systemic and inequitable access, where any pandemic or widespread vaccination response must be grounded in the principles of community, equity, transparency and Accountability, for example, all clinical research conducted in Africa implemented with resourced community engagement plans that span all phases of research, from protocol development to dissemination and access. Research must include women, transgender women, and other marginalized groups, such as people living with HIV.

A CLM approach like Ports2Arms is by no means a panacea, but its focus on identifying locally grounded realities and its ability to offer solutions through identifying barriers, highlighting enablers and using the data for evidence-based advocacy at grassroots community level, national and regional levels, and globally have the potential to take us much closer to achieving, at the least, the COVID-19 vaccination targets for the continent than the current strategies that have been employed, and lay a solid foundation for using similar approaches to guide future pandemic and emergency responses and related vaccination programmes.
